# Somewhat Egotistical

**Published:** 1904-01

**Authors:** 


### EDITORIAL NOTES AND COMMENTS.

The Business office of THE JOURNAL-RECORD is No. 1007-1008 Century Building.

The Editorial office is 318-319-320 The Prudential.

Address all Business communications to Dr. M. B. Hutchins, Mgr.

Make remittances payable to THE ATLANTA JOURNAL-RECORD OF MEDICINE.

On matters pertaining to the Editorial and Original Communications address Dr. Bernard Wolff, Atlanta.

Reprints of original articles will be furnished at cost price. Requests for the same should always be made *on the manuscript.*

We will present, post-paid, on request, to each contributor of an original article, twenty (20) marked copies of THE JOURNAL-RECORD containing such article.

### SOMEWHAT EGOTISTICAL.

We want to say a word about ourselves—always a subject of more importance to oneself than to the public, except in the case of a criminal or an actor or one who has achieved sufficient notoriety to arouse curiosity for intimate information. THE JOURNAL-RECORD is the result of the fusion of *The Atlanta Medical and Surgical Journal* and *The Southern Medical Record,* and after nearly five years of existence seems to demonstrate the truth of the German phrase *eintracht haelt macht,* or in union is strength.

We have witnessed the rise and fall of rival publications, coming up like a flower to be cut down and in the archives of past events we can read with the serene equanimity the legend *Hic jacet* on the simple head-stones of at least five aspirants which succumbed to marasmus or hydrocephalus or some kindred disorder directly affecting the nutrition or disturbing the balance. We have no desire to make merry at the discomfiture of others nor to point with taunting finger and a triumphant "I told you so." But we take it that the straw fires which left only blackened ashes served to illuminate the solid masonry of our own front, and to bring out in detail its burly strength. We attribute our success and prosperity, both of which have been admirably sustained during the past year, to honesty and straightforward dealing.

We have made no extravagant claims or pretenses that could not be established or sustained. We have been content to occupy a certain field and have not striven to go beyond it. This has been THE JOURNAL–RECORD'S policy, and we see no reason to change it in the future conduct of its affairs.

We have enjoyed the liberal patronage of high-class advertisers, as an inspection of the advertising pages will show. We have admitted no questionable advertisements, and when approached for rates for such have systematically ignored such requests. The literary department has been patronized by the best men in the State, and we have published papers from them and from others elsewhere of the highest practical and scientific value. We are not unmindful of the good will of our supporters, and we wish most earnestly to have them all display a personal interest in THE JOURNAL. The editorial columns are open to the profession as well as the other departments, and we invite those who so desire to avail themselves of this means of setting forth their views and opinions upon professional matters.

We wish for all of our friends continued prosperity in the year upon whose threshold we are now standing.

